# Development of Antibody Detection ELISA Based on Immunoreactive Toxins and Toxin-Derived Peptides to Evaluate the Neutralization Potency of Equine Plasma against *Naja atra* in Taiwan

**DOI:** 10.3390/toxins13110818

**Published:** 2021-11-19

**Authors:** Chien-Chun Liu, Yung-Chin Hsiao, Lichieh Julie Chu, Po-Jung Wang, Chien-Hsin Liu, Wen-Chin Hsieh, Jau-Song Yu

**Affiliations:** 1 Molecular Medicine Research Center, Chang Gung University, Tao-Yuan 33302, Taiwan; d000014743@cgu.edu.tw (C.-C.L.); hschin@mail.cgu.edu.tw (Y.-C.H.); julie.chu@mail.cgu.edu.tw (L.J.C.); d000016308@cgu.edu.tw (P.-J.W.); 2 Graduate Institute of Biomedical Sciences, College of Medicine, Chang Gung University, Tao-Yuan 33302, Taiwan; 3 Liver Research Center, Chang Gung Memorial Hospital, Linkou, Tao-Yuan 33305, Taiwan; 4 Center for Diagnostics and Vaccine Development, Centers for Disease Control, Ministry of Health and Welfare, Taipei 11561, Taiwan; liuch@cdc.gov.tw (C.-H.L.); vac@cdc.gov.tw (W.-C.H.); 5 Research Center for Food and Cosmetic Safety, College of Human Ecology, Chang Gung University of Science and Technology, Tao-Yuan 33303, Taiwan

**Keywords:** *Naja atra*, freeze-dried neurotoxic antivenom, neutralization potency, neurotoxin, epitope, peptide-based ELISA

## Abstract

*Naja atra*, also known as Taiwanese cobra, is one of the most prevalent venomous snakes in Taiwan. Clinically, freeze-dried neurotoxic antivenom (FNAV) produced from horses by Taiwan Centers for Disease Control (CDC) has been the only approved treatment for *N. atra* envenoming for the last few decades. During antivenom production, large numbers of mice are used in the in vivo assay to determine whether the neutralization potency of hyperimmunized equines is satisfactory for large-scale harvesting. However, this in vivo assay is extremely laborious, expensive, and significantly impairs animal welfare. In the present study, we aimed to develop an in vitro ELISA-based system that could serve as an alternative assay to evaluate the neutralization potency of plasma from hyperimmunized equines. We initially obtained 51 plasma samples with known (high or low) neutralization potency assessed in vivo from 9 hyperimmunized equines and subsequently determined their antibody titers against the five major protein components of *N. atra* venom (neurotoxin (NTX), phospholipase A2 (PLA_2_), cytotoxin (CTX), cysteine-rich secretory protein (CRISP), and snake venom metalloproteinase (SVMP)) via ELISA. The antibody titer against NTX was the most effective in discriminating between high and low potency plasma samples. To identify the specific epitope(s) of NTX recognized by neutralization potency-related antibodies, 17 consecutive NTX-derived pentadecapeptides were synthesized and used as antigens to probe the 51 equine plasma samples. Among the 17 peptides, immunoreactive signals for three consecutive peptides (NTX1-8, NTX1-9, and NTX1-10) were significantly higher in the high potency relative to low potency equine plasma groups (*p* < 0.0001). Our ELISA system based on NTX1-10 peptide (RWRDHRGYRTERGCG) encompassing residues 28–42 of NTX displayed optimal sensitivity (96.88%) and specificity (89.47%) for differentiating between high- and low-potency plasma samples (area under the receiver operating characteristic curve (AUC) = 0.95). The collective data clearly indicate that the antibody titer against NTX protein or derived peptides can be used to efficiently discriminate between high and low neutralization potency of plasma samples from venom-immunized horses. This newly developed antibody detection ELISA based on NTX or its peptide derivatives has good potential to complement or replace the in vivo rodent assay for determining whether the neutralization potency of equine plasma is satisfactory for large-scale harvesting in the antivenom production process against *N. atra.*

## 1. Introduction

Snakebite is a relatively neglected tropical disease affecting more than 1.8 million people worldwide annually [1]. In Taiwan, approximately 1000 snakebite cases occur each year predominantly by the six major venomous species, *Naja atra*, *Bungarus multicinctus*, *Trimeresurus stejnegeri*, *Protobothrops mucrosquamatus*, *Deinagkistrodon acutus,* and *Daboia russelii siamensis* [2,3]. *N. atra*, also known as Taiwanese cobra, has been classified as a category 1 World Health Organization (WHO) group in the guidelines for antivenom production [4], and accounts for 23.5–36% of envenoming cases in Taiwan [2,3].

In Taiwan, freeze-dried neurotoxic antivenom (FNAV) is available for clinical treatment of *N. atra* envenoming [5,6]. Taiwan Centers for Disease Control (CDC) have developed FNAV against *N. atra* and *B. multicinctus*, greatly facilitating control of snakebite-related mortality in Taiwan. During the process of antivenom production, horses are repeatedly immunized with snake venom and an in vivo murine assay is conducted to monitor the neutralization potency of hyperimmune horse plasma at different time-points. Once the neutralization potency reaches an acceptable level, the horse is bled for harvesting large-volume plasma for IgG purification and subsequent antivenom production. However, ethical considerations in the use of mice for preclinical testing of antivenom have been raised. The currently available in vivo neutralization assay seriously impairs the welfare of experimental rodents and large numbers of mice are sacrificed during the process of antivenom generation [7,8]. The possibility of implementing the 3R concept (Replacement, Reduction, and Refinement) in the manufacturing process and preclinical assessment of antivenom is therefore under serious consideration. Furthermore, the in vivo rodent assay currently employed to determine the neutralization potency of antivenom in the horse immunization process is extremely laborious, expensive, and time-consuming. The development of efficient alternative methods to replace (and/or reduce) animal testing in the process of antivenom production is therefore highly desirable [8,9].

In the past few decades, several ELISA studies attempting to assess the neutralization potency of antivenom have highlighted a significant correlation between the optical absorbance of ELISA and ED_50_ of in vivo murine testing [10,11,12,13,14,15]. While the potential of ELISA to serve as an alternative strategy for neutralization potency evaluation has been extensively investigated, this technique has not been applied for potency testing of clinically used antivenom, since animal model testing is still required for approval by the Food and Drug Administration. The applicability of ELISA in monitoring the neutralization potency of equine plasma following hyperimmunization to snake venom and determining whether the level of potency meets the criteria for harvesting during the process of antivenom manufacturing was investigated in the current study.

To develop ELISA for antivenom neutralization potency assessment, the selection of the appropriate antigens for detection is the most important step. Generally, antivenom is produced by immunizing horses with crude venom. However, some of the antibodies may be directed against non-toxic and more immunogenic components of snake venom, and therefore irrelevant for the neutralization potency. Therefore, using toxicity-related or potency-related protein components rather than the whole venom as antigens for ELISA would achieve better performance in determining the neutralization potency of antivenom [12,13]. Synthetic peptides that mimic potency-related epitopes on these toxic components can be effectively used as antigens and peptide-based ELISAs have been shown to outperform protein antigens in the evaluation of the neutralization potency [14]. Here, we examined two groups (high and low neutralization potency) of hyperimmunized equine plasma to identify potency-related proteins from *N. atra* venom and further explored the neutralization potency-related epitopes of these toxic proteins. Peptides corresponding to neutralization potency-related epitopes of target toxin proteins were used as antigens to develop ELISA capable of evaluating the neutralization potency of equine plasma in the antivenom manufacturing process.

## 2. Results

### 2.1. Neutralization Potency of Hyperimmunized Equine Plasma in a Rodent Model

The Tanaka unit assessment and related methods are routinely used to determine the neutralization potency of antivenom at the Centers of Disease and Control (CDC) in Taiwan. During the FNAV production process, 60 Tanaka units/mL for *N. atra* venom was defined as the acceptable potency of hyperimmunized equine plasma for large-scale harvesting. In total, 51 plasma samples from 9 hyperimmunized horses were utilized in our study. To assess the neutralization potency of each plasma sample, in vivo rodent testing was performed. Our data showed that 32 and 19 plasma samples had higher and lower neutralization potency than 60 Tanaka units/mL, respectively. The two sample groups were categorized as “High Potency” and “Low Potency”, respectively (Supplementary Table S1).

### 2.2. Preparation and Optimization of ELISA Assays for Equine Antibody Detection

**Figure 1:**
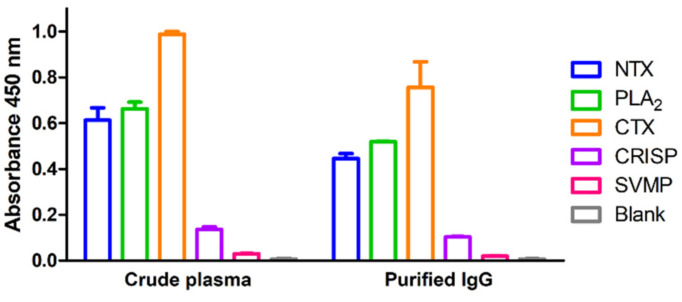
Immunorecognition of five toxic proteins of *N. atra* venom by hyperimmunized horse plasma and caprylic acid-purified IgG. Each of the five snake venom proteins was coated on a 96-well ELISA plate (10 ng/well), and crude equine plasma (1:20,000 dilution) and caprylic acid-purified IgG (1:20,000 dilution) with high neutralization potency applied to develop ELISA signals. Each data point represents mean ± SD of triplicate experiments.

Five dominant protein components of *N. atra* venom, specifically, neurotoxin (NTX), phospholipase A2 (PLA_2_), cytotoxin (CTX), cysteine-rich secretory protein (CRISP), and snake venom metalloproteinase (SVMP), were prepared using RP-HPLC as described previously [16]. To optimize the concentrations of antigens (venom components) and antibody (equine plasma) for ELISA, a checkerboard titration analysis was performed. The optimal concentration of venom antigens was determined as 10 ng/well and the optimal plasma dilution as 1:20000. Higher concentrations of coated antigens or lower plasma dilutions resulted in saturated signals in positive high-titer plasma and unacceptable background in negative controls, which, in turn, decreased the signal-to-noise ratio. Under optimal conditions of established ELISA, the optical density (OD) values of a randomly selected, high-potency plasma sample (batch no. 38) for the five major venom components varied significantly (from <0.1 to ~1.0). The immunorecognition signals against CTX, PLA_2_, and NTX were considerably higher than those against CRISP and SVMP (Figure 1, left panel).

**Figure 2:**
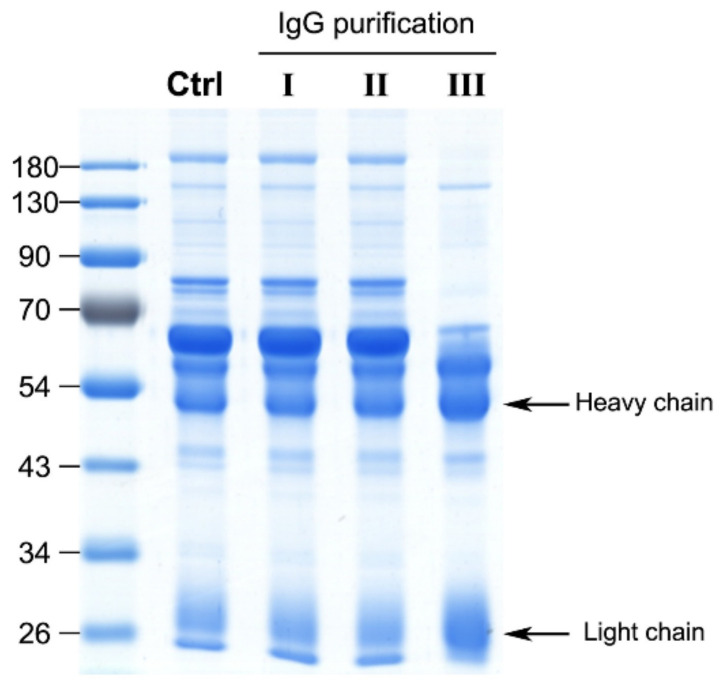
Performance of different IgG purification strategies. Three strategies were applied to purify IgGs from a hyperimmunized horse plasma sample, including ultrafiltration with a Vivaspin 500 concentrator (molecular weight cut-off: 100 kDa) (strategy I), an Amicon Ultra 0.5 mL device (molecular weight cut-off: 100 kDa) (strategy II) and caprylic acid precipitation (strategy III). Purified fractions were analyzed via SDS-PAGE (10 μg protein/lane) and further visualized with Coomassie blue staining. The control (Ctrl) lane denotes the protein profile of unprocessed horse plasma. The arrows highlight the heavy and light chains of IgG.

Generally, crude horse plasma was used to determine the neutralization potency in the antivenom manufacturing process; however, unlike in vivo rodent assays, unprocessed plasma may have a matrix effect in in vitro ELISA assay. To assess the contribution of plasma matrix to the ELISA signal, equine IgG in plasma was purified using caprylic acid and ultrafiltration devices. Two ultrafiltration devices, Vivaspin 500 and Amicon Ultra with 100 kDa cut-off were selected for testing. As shown in Figure 2, caprylic acid purification outperformed the ultrafiltration devices in the enrichment of IgGs from crude plasma samples, as evident from sodium dodecyl sulfate polyacrylamide gel electrophoresis (SDS-PAGE) analysis of the protein profiles. Next, caprylic acid-purified IgGs were used to detect the five major venom components and the results were compared with those obtained using crude plasma sample (Figure 1, right panel). Our analysis revealed a similar tendency of crude plasma and caprylic acid-purified IgGs in recognizing the five major venom components, although ELISA signals against all venom proteins became lower after purification. This finding indicates that limited IgG is lost in the process of caprylic acid purification, suggesting that the plasma matrix does not affect the signal or contribute to noise in our ELISA assay for equine antibody detection. Accordingly, we selected crude plasma samples for ELISA in subsequent experiments.

### 2.3. Selection of Immunoreactive Venom Proteins for Differentiating the Neutralization Potency Levels of Equine Plasma

**Figure 3:**
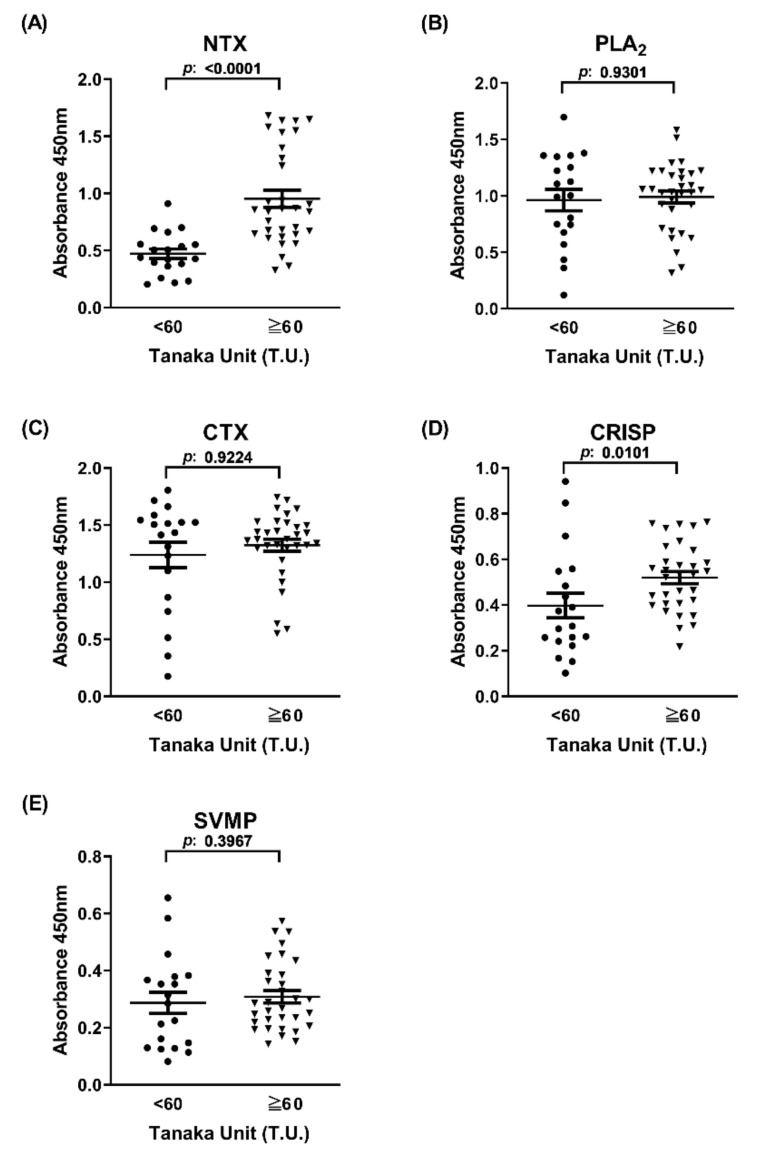
ELISA signals of the two horse plasma groups towards five cobra venom components. Horse plasma samples (high potency (Tanaka units (T.U.) ≥ 60, n = 32) and low potency (T.U. < 60, n = 19)) were subjected to ELISA against (**A**) NTX, (**B**) PLA_2_, (**C**) CTX, (**D**) CRISP, and (**E**) SVMP. ELISA signals for individual plasma samples were analyzed and presented as dot plots. Each data point represents mean of duplicate experiments. The horizontal line denotes the mean value of ELISA signals in each group.

To evaluate the potential of the ELISA signal derived from venom components in differentiating between high and low neutralization potency of equine plasma, the immunorecognition capability of 51 equine plasma samples towards the five major venom proteins was examined. As shown in Figure 3, ELISA signals against NTX and CRISP, but not PLA_2_, CTX, and SVMP, were able to discriminate between high-potency and low-potency groups of equine plasma (*p* < 0.05). NTX-based ELISA presented the highest potential (~2-fold difference in ELISA signals between groups, *p* < 0.0001) in differentiating between the two equine plasma groups (Figure 3A). Notably, although the average ELISA signal of the 51 plasma samples towards CTX was highest among the five antigens, CTX-based ELISA signals were not associated with neutralization potency (Figure 3C).

**Figure 4:**
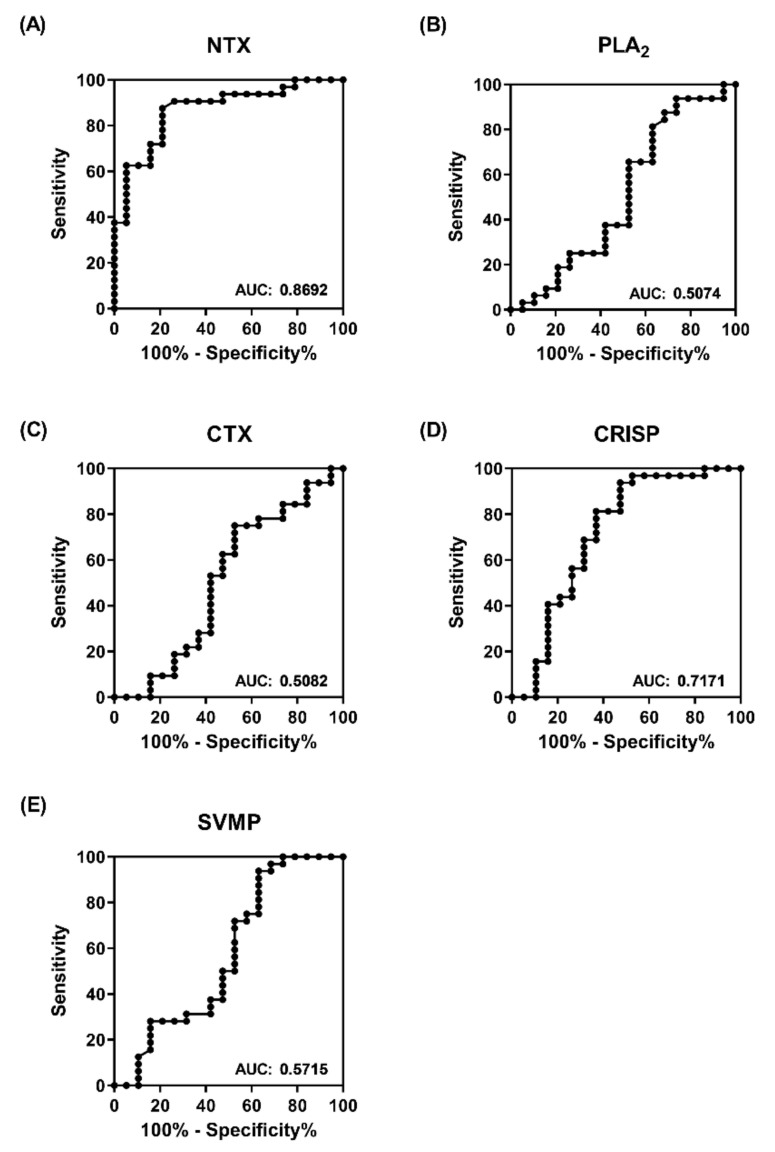
Power of protein-based ELISA for discriminating high-potency from low-potency equine plasma. ROC curves of ELISA signals towards (**A**) NTX, (**B**) PLA_2_, (**C**) CTX, (**D**) CRISP, and (**E**) SVMP for differentiating between high- and low-potency plasma. The value of the area under the curve (AUC) denotes the power of target analytes in the classification of high- and low-potency horse plasma.

Next, we applied receiver operating characteristic (ROC) curve analysis to estimate the sensitivity and specificity of venom protein-based ELISA in discriminating between the two equine plasma groups. As shown in Figure 4, the area under the curve (AUC) for NTX-, PLA_2_-, CTX-, CRISP-, and SVMP-based ELISA was estimated as 0.87, 0.5, 0.5, 0.72, and 0.57, respectively. The results clearly support the superiority of NTX-based ELISA over the other four antigens and the AUC value of 0.87 was considered excellent in terms of discriminatory power with high accuracy (Figure 4A) [17]. At the optimal cut-off value of 0.55, sensitivity and specificity were determined as 87.5% and 78.95%, respectively. Thus NTX-based ELISA for antibody detection presents an effective alternative strategy to establish whether the neutralization potency of equine plasma is satisfactory for large-scale harvesting.

### 2.4. Identification of Neutralization Potency-Related Epitopes in NTX

**Figure 5:**
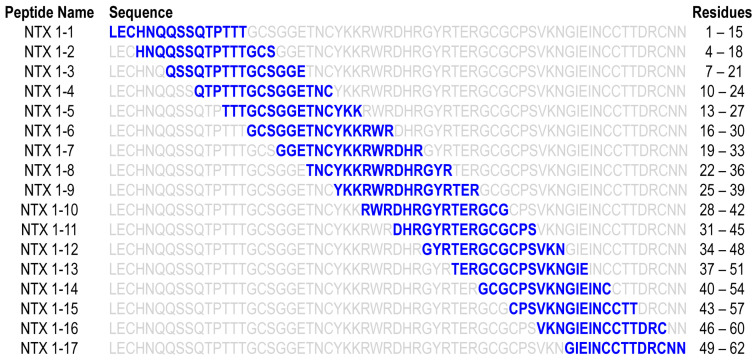
Scheme for synthesis of NTX-derived peptides. Each NTX-derived peptide is highlighted in blue and the relative positions of peptides within the overall amino acid sequence of NTX are illustrated. In total, 17 peptides encompassing the entire sequence of NTX were synthesized.

Our data showed that the NTX-specific antibody titer is significantly and positively associated with the neutralization potency of plasma samples from hyperimmunized horses. Specific epitopes on NTX are assumed to be responsible for recognition by antibodies with neutralization potency in hyperimmunized horse plasma samples. To identify these epitopes in NTX, we synthesized 17 NTX-derived peptides including all possible linear epitopes (Figure 5). Each peptide had 15 residues and was overlapped and frameshifted by three residues with the consecutive sequence. The 17 pentadecapeptides encompassed the entire amino acid sequence of NTX.

**Figure 6:**
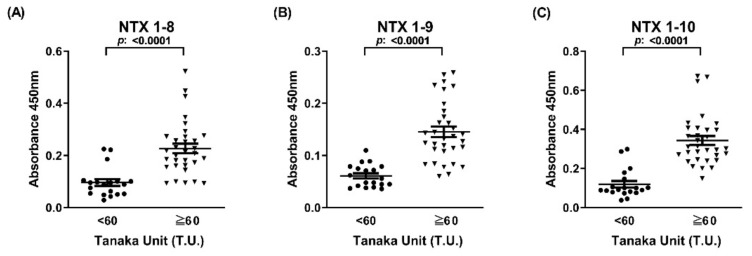
ELISA signals of the two horse plasma groups towards three NTX-derived peptides. Horse plasma samples (high-potency group (T.U. ≥ 60, n = 32) and low-potency group (T.U. < 60, n = 19)) were subjected to ELISA against (**A**) NTX 1-8, (**B**) NTX 1-9, and (**C**) NTX 1-10, respectively. ELISA signals for individual plasma samples were analyzed and presented as dot plots. Each data point represents mean of duplicate experiments. The horizontal line denotes the mean value of ELISA signals in each group.

The 17 peptides were coated onto ELISA plates and used to probe titers of peptide-specific antibodies in the 51 equine plasma samples. Immunoreactive signals towards three consecutive peptides (NTX1-8, NTX1-9, and NTX1-10) were significantly higher in the high-potency equine plasma group relative to the low-potency group (*p* < 0.0001) (Figure 6). Regarding the other 14 peptides, ELISA signals were either not significantly different between the two equine plasma groups (NTX1-6, NTX1-7, NTX1-11, NTX1-12, NTX1-14, NTX1-15, NTX1-16, and NTX1-17) or markedly lower in the high-potency equine plasma group (NTX1-1, NTX1-2, NTX1-3, NTX1-4, NTX1-5, and NTX1-13) (Supplementary Figure S1). Although the latter six peptides also displayed the capability to discriminate between the two groups, their ELISA signals were very weak (<0.1) and negatively associated with the neutralization potency of equine plasma, thus precluding a potential role as neutralization potency-related epitopes of NTX.

**Figure 7:**
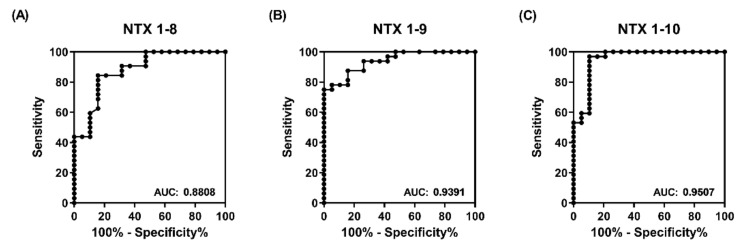
Power of peptide-based ELISA for discriminating high-potency from low-potency plasma groups. ROC curves of ELISA signals towards (**A**) NTX 1-8, (**B**) NTX 1-9, and (**C**) NTX 1-10 for differentiating between high- and low-potency plasma samples.

Next, ROC curve analysis was applied to calculate the sensitivity and specificity of NTX peptide-based ELISA in discriminating between the two equine plasma groups. As shown in Figure 7, AUC values of ELISA based on NTX1-8, NTX1-9, and NTX1-10 were determined as 0.881, 0.939, and 0.951, respectively, considered outstanding discriminatory power with high accuracy. NTX1-10 presented the highest AUC value (0.951) and at the optimal cut-off value of 0.2, sensitivity and specificity were determined as 96.88% and 87.47%, respectively (Figure 7C). The AUC values of the other 14 peptides, ranging from 0.503 to 0.879, are shown in Supplementary Figure S2.

### 2.5. Location of Neutralization Potency-Related Epitopes in the Loop II Domain of NTX

**Figure 8:**
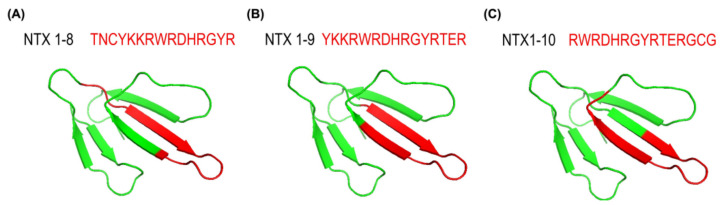
Three-dimensional structure of NTX protein (P60770) from *N. atra*. The structure image was created using Mol* Viewer (https://molstar.org/viewer/, accessed on 15 November 2021). The relative positions of (**A**) NTX1-8, (**B**) NTX1-9, (**C**) NTX1-10 in the NTX homology model are depicted in red.

To further explore the spatial positions of the three peptides (NTX1-8, NTX1-9, and NTX1-10) within NTX protein, we retrieved the 3D protein structure of NTX from the Protein Data Bank (https://www.rcsb.org/, accessed on 15 November 2021) PDB code: 1COE) [18] for mapping of the three peptides. Interestingly, all three peptides encompassing residues 22–42 were located in the loop II domain of NTX (Figure 8), an essential region for acetylcholine receptor binding [19,20]. Our results indicate that the loop II domain of NTX contains important epitope(s) related to the neutralization potency of equine plasma against *N. atra* venom and support the potential of NTX1-10 peptide-based ELISA in determining whether the neutralization potency of plasma samples from hyperimmunized horses fulfills the criteria for large-scale bleeding for antivenom production, which could present an effective alternative to in vivo rodent testing in the process of antivenom production.

## 3. Discussion

In vivo murine model assays are currently used for determining the neutralization potency of antivenom products under development and monitoring the immune response of horses after continued immunization. These crucial assays are laborious, expensive, and require a large number of experimental mice to reduce intra-assay variability. In order to limit the cost and prevent unnecessary suffering of animals, various in vitro surrogate assays, such as ELISA, competitive ELISA [21], agglutination assay [22], receptor binding assay [23,24], and enzyme activity assay [25], have been explored as potential alternatives for potency estimation. ELISA appears the most promising alternative since it is a simple and universal method that can be applied to all venom types, unlike the receptor binding assay that is specifically designed for *Elapidae* snake venom neurotoxins. Furthermore, ELISA can be easily established in most laboratories and can provide quantitative and reproducible results for the estimation of neutralization potency. However, several concerns have been raised about the practical utility of this in vitro immunoassay, which produces results that differ from those of the in vivo neutralization assay, although they are highly correlated. A number of immunoreactive venom antigens and antigenic epitopes recognized by hyperimmunized equine plasma may not be relevant for venom lethality in mice, leading to inconsistent results between neutralization potency and antibody-detected ELISA signals [26]. To address this issue, the selection of toxic components and measurement of the immunoreactivity of antibodies specifically against these components would be a more appropriate strategy than using the whole venom as antigens to develop useful ELISAs for estimating the neutralization potency of equine plasma [11]. Moreover, these toxicity-related proteins have binding or catalytic domains that contribute to their biological and toxicological functions. Synthesis of peptides mimicking these domains that may serve as alternative toxic components for ELISA analysis should not only enhance specificity and sensitivity of evaluation of the neutralization potency of equine plasma but also reduce the cost of assay development.

Using RP-HPLC-isolated toxic component fractions as screening antigens, we identified three protein families (CTX, PLA_2_, and NTX) as immunoreactive antigens highly recognized by hyperimmunized equine plasma (Figure 1). CTX was the most dominant protein in *N. atra* venom providing the strongest immunoreactive signal. However, the signal was not correlated with neutralization potency (Figure 3C), indicating that CTX is not a suitable target for the development of a neutralization potency assay. Conversely, while the relative abundance of NTX was the lowest among the three immunoreactive antigens [16], the ELISA signal of antibodies against NTX was highly correlated with neutralization potency results in our rodent model. Based on these results, NTX was determined as a major toxic protein inducing lethality in mice despite lower relative abundance than CTX and PLA_2_ in whole *N. atra* venom.

NTX of *N. atra* venom, also known as cobrotoxin, is a type I (short-chain) alpha neurotoxin from a three-finger toxin family that serves as an antagonist of muscle nicotinic acetylcholine receptor (nAChR) to inhibit neuromuscular transmission [27]. Loop II of the three-finger toxin structure highly conserved within this protein family is the binding domain of nAChR [18,28,29,30]. Moreover, the loop II domain of NTX has been determined as one of the major immunoreactive epitopes in *N. atra* venom recognized by FNAV [31]. Consistent with this observation, NTX1-8, NTX1-9, and NTX1-10 were identified in the loop II domain of NTX in the current study. Moreover, the common motif of these three NTX-derived peptides, RWRDHRGYR, was located precisely at the top region of loop II (Figure 8). Antibody titers against these epitopes were correlated with neutralization potency (Figure 6) and the antibody-specific ELISA signals based on these NTX-derived peptides were effectively used to determine the adequacy of the immune response status of target horses for large-scale bleeding. These results are consistent with our hypothesis that measurement of antibodies against specific binding motifs or functional domains of toxicity-related proteins could be used to precisely estimate the neutralization potency levels in equine plasma.

## 4. Conclusions

The antibody titer against NTX or derived peptides could be used to efficiently discriminate between plasma samples from venom-immunized horses with high and low neutralization potency. Our newly developed antibody detection ELISA based on the NTX-derived peptide, RWRDHRGYRTERGCG, displayed optimal power (96.88% sensitivity and 89.47% specificity) for determining whether the neutralization potency of equine plasma is satisfactory for large-scale harvesting. NTX peptide-based ELISA has the potential to complement or replace the in vivo rodent assay in the production process of antivenom against *N. atra.* While in vitro assays are highly valuable in deciding the time-point of the blood draw and selecting batches of plasma to be processed, in vivo assays are still required to be performed before the final antivenom product came to market.

## 5. Materials and Methods

### 5.1. Snake Venom and Hyperimmunized Horse Plasma

Lyophilized venom of *N*. *atra* was obtained from the Centers of Disease and Control Taipei, Taiwan. The venom was freeze-dried and stored at −20 ℃ before use. Neurotoxic venom (*B*. *multicinctus* and *N*. *atra*)-immunized horse plasma was provided by the Centers of Disease and Control, Taipei, Taiwan. In total, 51 batches of plasma samples were collected from 9 hyperimmunized horses at different time-points (Supplementary Table S1) and stored at −80 ℃ before experimental use.

### 5.2. Venom Protein Components and Protein-Derived Peptides

Five major protein components of *N. atra* venom were isolated via reverse-phase high-performance liquid chromatography (RP-HPLC) [16]. Synthetic peptides corresponding to the sequences of target proteins were designed based on the following criteria: (1) overlapping 15-mer peptides, (2) frameshift of three residues, and (3) spanning entire sequences of proteins without considering the signal peptide for synthesis. All protein-derived peptides were synthesized by Kelowna International Scientific Inc (Taipei, Taiwan).

### 5.3. Purification of IgG from Horse Plasma

Two strategies were used for IgG purification, specifically, caprylic acid precipitation and ultrafiltration. Caprylic acid precipitation was performed following a previously described protocol [32]. Briefly, 100 μL horse plasma was diluted with 200 μL of 60 mM sodium acetate (pH 4.6) and 6 μL of caprylic acid added dropwise into the solution with continuous stirring for 30 min. The mixture was centrifuged at 5000× *g* at 4 °C for 30 min. The supernatant with IgG was dialyzed against phosphate buffered saline (PBS) and concentrated to 100 μL in PBS. For ultrafiltration, 100 μL horse plasma was filtered using Vivaspin 500 centrifugal concentrators (Sartorius, Göttingen, Germany) and an Amicon Ultra 0.5 mL device (Millipore, Burlington, MA, USA) according to the manufacturer’s instructions.

### 5.4. Determination of the Neutralization Potency of Horse Plasma in Tanaka Units

The neutralization potency of antivenom in Taiwan was estimated as Tanaka units/mL as described previously [33]. The routine method used to determine the neutralization potency of the hyperimmunized horse is acceptable for large-scale harvesting. Briefly, the minimal lethal dose (MLD) of *N. atra* venom was determined as 13 μg, which displayed the lowest dose of venom-inducing lethality in all injected mice. Half a milliliter of 4 MLD of *N. atra* venom was mixed with 0.5 mL of 3-fold diluted horse plasma and incubated for 1 h at 37 °C. ICR mice (n = 3) were subcutaneously injected with 0.2 mL of the mixture and survival was recorded at 24 h post-injection. In conditions where all the injected mice survived, the hyperimmunized horse was acceptable for bleeding, with an estimated neutralization potency higher than or equal to 60 Tanaka units/mL. Neutralization potency was defined as <60 Tanaka units/mL at survival rates of <100%.

### 5.5. Experimental Animals and Ethics Statement

Experiments were performed on 3 week-old littermate male mice (ICR strain). Mice were maintained in specific pathogen-free conditions under a 12:12 h light-dark cycle at a temperature of 22 °C and humidity level of 60–70%. Animals had *ad libitum* access to food and water. Experiments involving the care and injection of mice with various venom types and horse plasma were reviewed and approved by the Institutional Animal Care and Use Committee of Chang Gung University (Permit Number: CGU109-095). The protocol of the animal study on mice was based on the guidelines provided by the Council for International Organizations of Medical Sciences (CIOMS) [34].

### 5.6. Antibody Detection ELISA

Cobra venom proteins (10 ng) or NTX-derived peptides (100 ng) were diluted in 50 μL PBS and coated onto 96-well polystyrene clear microplates (Corning Inc., Corning, NY, USA) with incubation at 4 ℃ overnight. Plates were washed six times with 100 μL phosphate-buffered saline containing 0.05% Tween-20 (PBST) and blocked with 200 μL of 1% ovalbumin in PBS at room temperature for 1 h. After washing wells with PBST six times, horse plasma was diluted (1:20,000) and added to each well, followed by incubation of the plate at room temperature for 1 h. After six washes with PBST, rabbit anti-horse IgG conjugated with horseradish peroxidase (HRP) (Bethyl Laboratories, Montgomery, TX, USA) was added to each well and incubated at room temperature for 1 h. Plates were further washed six times with PBST and 50 μL of tetramethylbenzidine (TMB) substrate (Clinical Science Products Inc., Mansfield, MA, USA) was added to each well for 10 min. The reaction was terminated with 25 μL of 2N H_2_SO_4_ (J.T Baker, Radnor, PA, USA) and absorbance of each well measured with a SpectraMax M5 microplate reader (Molecular Devices, San Jose, CA, USA) at excitation and emission wavelengths of 450 and 540 nm, respectively. Each assay was performed in duplicate, and the mean of absorbance value was used for further statistical analysis.

### 5.7. Statistical Analysis

Statistical analysis was performed using unpaired t-tests. Both unpaired t-test and receiver operating characteristic (ROC) curve analysis were performed using GraphPad Prism 5 software (GraphPad Software, San Diego, CA, USA). Differences were considered statistically significant at *p*-values < 0.05.

## Data Availability

The data presented in this study are available on request from the corresponding author.

## Supplementary Materials

The following are available online at https://www.mdpi.com/article/10.3390/toxins13110818/s1, Figure S1: ELISA signals for 14 NTX-derived peptides of horse plasma samples with different potencies., Figure S2: ROC curves of ELISA signals for 14 NTX-derived peptides differentiating between high- and low-potency plasma samples., Table S1: Detailed description of hyperimmunized equine antiserum.
