# Supplementary material for: Development of Antibody Detection ELISA Based on Immunoreactive Toxins and Toxin-Derived Peptides to Evaluate the Neutralization Potency of Equine Plasma against Naja atra in Taiwan
Source: Toxins (Basel). 2021 Nov 19;13(11):818. doi: 10.3390/toxins13110818 (PMC8622849; doi:10.3390/toxins13110818)
Supplement: Supplementary file 1 [file toxins-13-00818-s001.zip › toxins-1443319-supplementary.pdf]

toxins

MDPI

Supplementary Information

Development of Antibody Detection ELISA Based on Immunoreactive Toxins and Toxin-Derived Peptides to Evaluate the Neutralization Potency of Equine Plasma against *Naja atra* in Taiwan

Chien-Chun Liu [1], Yung-Chin Hsiao [1,2,3], Lichieh Julie Chu [1,2,3], Po-Jung Wang [1], Chien-Hsin Liu [4], Wen-Chin Hsieh [4] and Jau-Song Yu [1,2,3,5,*]

[1] Molecular Medicine Research Center, Chang Gung University, Tao-Yuan 33302, Taiwan; d000014743@cgu.edu.tw (C.-C.L.), hschin@mail.cgu.edu.tw (Y.-C.H.), julie.chu@mail.cgu.edu.tw (L.J.C.), d000016308@cgu.edu.tw (P.-J.W.)
[2] Graduate Institute of Biomedical Sciences, College of Medicine, Chang Gung University, Tao-Yuan 33302, Taiwan
[3] Liver Research Center, Chang Gung Memorial Hospital, Linkou, Tao-Yuan 33305, Taiwan.
[4] Center for Diagnostics and Vaccine Development, Centers for Disease Control, Ministry of Health and Welfare, Taipei 11561, Taiwan; liuch@cdc.gov.tw (C.-H.L.); vac@cdc.gov.tw (W.-C.H.)
[5] Research Center for Food and Cosmetic Safety, College of Human Ecology, Chang Gung University of Science and Technology, Tao-Yuan 33303, Taiwan
* Correspondence: yusong@mail.cgu.edu.tw; Tel.: +886-3-2118800 (ext. 5171); Fax: +886-3-2118891

Table S1. Detailed description of hyperimmunized equine antiserum.

Batch No.	Horse No.	Sampling Date	Neutralization Titer (T.U.)	Defined Group
1	351	2018/11/13	<60	Low-Potency
2	354	2020/3/17	<60	Low-Potency
3	356	2019/11/5	<60	Low-Potency
4	356	2020/3/17	<60	Low-Potency
5	356	2020/3/31	<60	Low-Potency
6	356	2020/5/26	<60	Low-Potency
7	401	2019/12/30	<60	Low-Potency
8	401	2020/2/4	<60	Low-Potency
9	401	2020/3/3	<60	Low-Potency
10	401	2020/3/31	<60	Low-Potency
11	406	2018/4/17	<60	Low-Potency
12	406	2018/6/26	<60	Low-Potency
13	407	2020/3/17	<60	Low-Potency
14	418	2019/12/30	<60	Low-Potency
15	418	2020/2/4	<60	Low-Potency
16	418	2020/3/3	<60	Low-Potency
17	418	2020/3/31	<60	Low-Potency
18	420	2019/12/30	<60	Low-Potency
19	420	2020/1/14	<60	Low-Potency
20	354	2018/11/27	≥60	High-Potency
21	354	1018/12/11	≥60	High-Potency
22	354	2019/5/7	≥60	High-Potency
23	354	2019/5/21	≥60	High-Potency
24	354	2019/10/22	≥60	High-Potency
25	354	2019/11/5	≥60	High-Potency
26	354	2020/3/31	≥60	High-Potency
27	354	2020/4/14	≥60	High-Potency
28	354	2020/4/28	≥60	High-Potency

29	354	2020/5/12	≥60	High-Potency
30	354	2020/5/26	≥60	High-Potency
31	356	2019/4/23	≥60	High-Potency
32	356	2019/5/21	≥60	High-Potency
33	356	2019/10/22	≥60	High-Potency
34	356	2020/4/14	≥60	High-Potency
35	356	2020/4/28	≥60	High-Potency
36	356	2020/5/12	≥60	High-Potency
37	366	2018/11/13	≥60	High-Potency
38	407	2018/4/17	≥60	High-Potency
39	407	2019/4/23	≥60	High-Potency
40	407	2019/5/7	≥60	High-Potency
41	407	2019/5/21	≥60	High-Potency
42	407	2019/10/22	≥60	High-Potency
43	407	2019/11/5	≥60	High-Potency
44	407	2020/3/31	≥60	High-Potency
45	407	2020/4/14	≥60	High-Potency
46	407	2020/4/28	≥60	High-Potency
47	407	2020/5/12	≥60	High-Potency
48	407	2020/5/26	≥60	High-Potency
49	420	2020/2/4	≥60	High-Potency
50	420	2020/3/3	≥60	High-Potency
51	420	2020/3/17	≥60	High-Potency

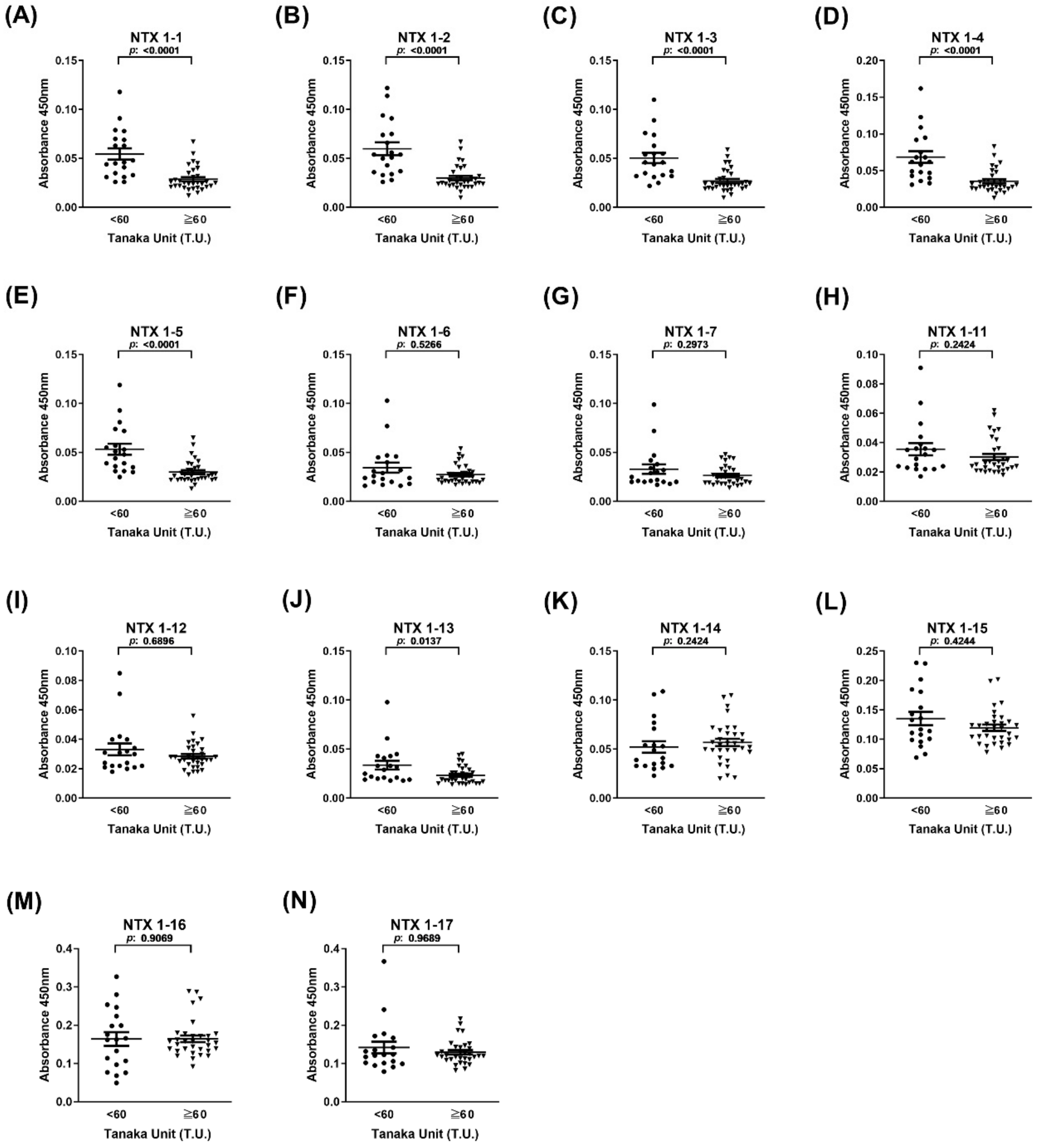

Figure S1. ELISA signals for 14 NTX-derived peptides of horse plasma samples with different potencies.

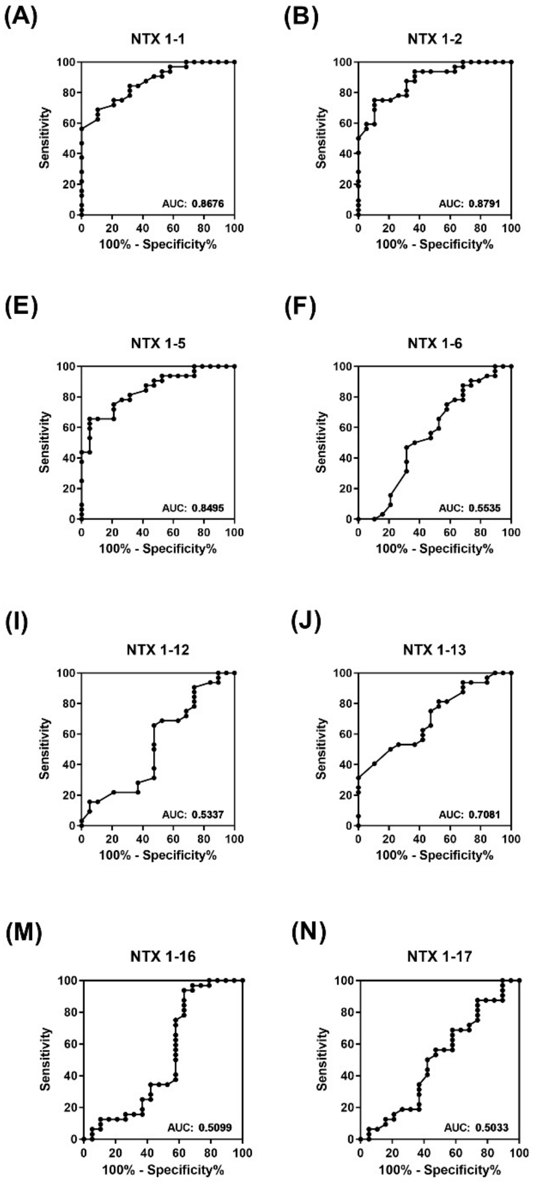

Figure S2. ROC curves of ELISA signals for 14 NTX-derived peptides differentiating between high- and low-potency plasma samples.
